# Upscaling Inverted Perovskite Solar Cells: Optimization of Laser Scribing for Highly Efficient Mini-Modules

**DOI:** 10.3390/mi11121127

**Published:** 2020-12-20

**Authors:** Francesco Di Giacomo, Luigi A. Castriotta, Felix U. Kosasih, Diego Di Girolamo, Caterina Ducati, Aldo Di Carlo

**Affiliations:** 1 Centre for Hybrid and Organic Solar Energy (CHOSE), Department of Electronic Engineering, University of Rome Tor Vergata, 00133 Rome, Italy; luigi.angelo.castriotta@uniroma2.it; 2 Department of Materials Science and Metallurgy, University of Cambridge, 27 Charles Babbage Road, Cambridge CB3 0FS, UK; fuk21@cam.ac.uk (F.U.K.); cd251@cam.ac.uk (C.D.); 3 Department of Chemical Materials and Production Engineering, University if Naples Federico II, Piazzale Tecchio 80, Fuorigrotta, 80125 Naples, Italy; diego.digirolamo91@gmail.com; 4 LASE–Laboratory for Advanced Solar Energy, National University of Science and Technology MISiS, Leninsky Ave. 6, 119049 Moscow, Russia

**Keywords:** perovskite solar cells, laser ablation, perovskite solar module

## Abstract

The upscaling of perovskite solar cells is one of the challenges that must be addressed to pave the way toward the commercial development of this technology. As for other thin-film photovoltaic technologies, upscaling requires the fabrication of modules composed of series-connected cells. In this work we demonstrate for the first time the interconnection of inverted modules with NiO_x_ using a UV ns laser, obtaining a 10.2 cm^2^ minimodule with a 15.9% efficiency on the active area, the highest for a NiO_x_ based perovskite module. We use optical microscopy, energy-dispersive X-ray spectroscopy, and transfer length measurement to optimize the interconnection. The results are implemented in a complete electrical simulation of the cell-to-module losses to evaluate the experimental results and to provide an outlook on further development of single junction and multijunction perovskite modules.

## 1. Introduction

The research on perovskite photovoltaics gained great momentum in the past 10 years. Major improvements in the efficiency and stability of perovskite solar cells (PSCs) have been achieved, making this technology the most efficient and promising in the class of thin-film photovoltaics (PV). Record small area devices have achieved certified power conversion efficiencies (PCEs) of 25.5% lately [1]. The versatility of PSCs is reflected by the variety of architectures that have been reported. We can identify two main classes: n-i-p and p-i-n cells. The former starts with an electron transport layer (ETL) over the transparent conductive oxide (TCO) and so far has been exhibiting relatively higher PCEs [2]. The p-i-n stack (also called inverted) reverses the order of the layers: it starts with a hole transport layer (HTL) on top of the TCO, followed by the perovskite layer, an electron transport layer (ETL), and a top electrode. It is often reported to be more stable and can be made without expensive HTL or top electrode materials (e.g., spiro-OMeTAD or Au often used in n-i-p PSCs) [3]. These advantages make it a more viable candidate for the development of commercial products, but the research on large-area perovskite devices is still mostly focused on the n-i-p architecture. Recently, the development of large-area p-i-n devices has been accelerated by the successful implementation of polytriarylamine (PTAA) as an HTL [4,5,6], while there are only a few reports on large-area inverted PSCs using a NiO_x_ HTL [7,8,9].

Besides the deposition of the layers over large areas, the upscaling of thin-film PV requires the development of monolithic interconnection to fabricate series-connected modules (if one excludes the use of Si/thin-film tandem). The need for the series interconnection arises from a series of arguments. The layers required for any thin-film PV technologies, i.e., cadmium telluride (CdTe), copper indium gallium sulfide/selenide (CIGS/Se), microcrystalline silicon (μc-Si) or amorphous silicon (a-Si), as well as organic PV and perovskite PV, can be deposited on large-sized substrates (i.e., in the m^2^ range). This advantage eliminates the additional costs and area losses required by the series connection of Si wafers used in crystalline silicon PV. At least one of the electrodes of a PV device (excluding interdigitated back-contact cells, an uncommon choice for thin-film PV) is based on a TCO to allow the collection of the incident light [10]: this layer cannot be too thick to maintain a good degree of transparency, consequently limiting the sheet resistance to values in the order of 7 to 60 Ω □^−1^ [11]. While the resistivity of the TCO could be negligible for small area devices (<1 cm^2^), this is not the case for large area ones. There are two options to mitigate the resistive losses: either the use of a current-collecting metal grid as for Si wafers or to divide the module into multiple series-connected cells. A metal grid is well-suited for Si wafers but will not be sufficient for devices in the scale of square meters (a 2 m^2^ module with 25 mA cm^−2^ current density output would generate 500 A of current). Since the layers of a thin-film device are deposited over a substrate (glass sheet, polymer foil, or metal foil), they can be patterned during their deposition (i.e., by shadow masks for vacuum deposition or by printing techniques for solution processing) or afterwards (by laser ablation or mechanical scribing). Using one of these methods, it is possible to pattern the layers to fabricate a module composed of series-connected cells. In this arrangement, the module’s output current is limited to that of one subcell, drastically decreasing the resistive losses and removing the need for current-collecting grids. This method is the most frequently used to upscale a thin-film solar cell and PSCs are no exception [12,13]. The voltage of the module will be, ideally, the sum of the subcells‘ voltages, while the current of the module will be limited by the lowest current produced by any of the subcells. Therefore, developing a homogeneous deposition process for the functionals layers is essential to maximize the module performance.

**Figure 1:**
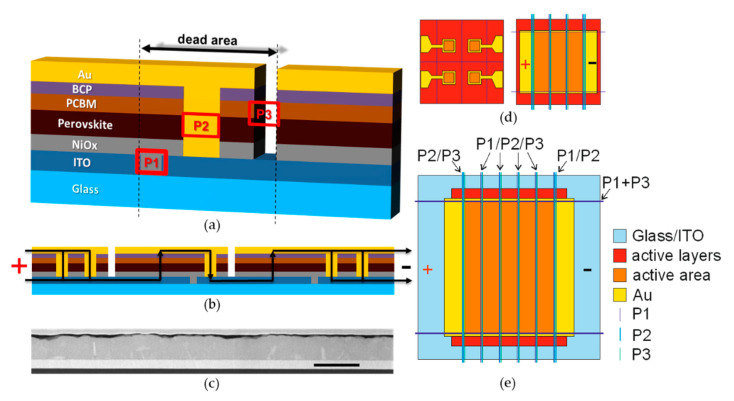
(**a**) A scheme of the pin architecture used and of the P1 P2 P3 interconnection; (**b**) A scheme of the charge flow in the series connection among cells and toward the contact; (**c**) Cross-sectional STEM-HAADF image of the stack used (scale bar is 1 µm); (**d**) design of the 0.09 cm^2^ cell and of the 2.25 cm^2^ modules; (**e**) Design of the 10.2 cm^2^ module.

The most common method employed to realize the interconnection of such an architecture is called P1-P2-P3 process. The P1 process insulates the bottom electrode of neighboring cells. The P2 patterning step selectively removes a slice of the entire layer stack, except TCO, between adjacent cells to permit their series connection through subsequent deposition of the top electrode. Finally, the P3 step is applied to separate the just-realized top electrode layer. An interconnection scheme (Figure 1, top left) is constituted by some active zones that are dedicated to the photogeneration of electric charges and, thus, to the photovoltaic energy conversion, and dead zones, dedicated to the interconnection between adjacent cells. It is possible to define the Active Area (AA) as that part of the substrate dedicated to the photovoltaic energy conversion and the Dead Area (DA), as the part where the interconnection of cells takes place, and which does not contribute to the energy conversion. The DA is formed by the P1, P2 and P3 zones and by two safety areas that are interposed between them. The ratio between the photoactive area (AA) and the aperture area of the module (AR, the sum of active and dead area) is usually referred to as the geometric fill factor (GFF).

**Figure 2:**
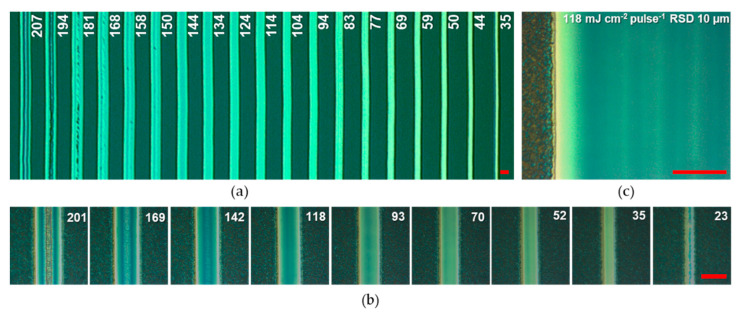
(**a**) Optical microscope image of a P2 power scan with a 355 UV ns laser over a sample made of glass/ITO/NiO_x_/perovskite/PCBM/BCP. The numbers indicate the fluence per pulse in mJ cm^−2^. Given the scribing speed of 195 mm s^−1^ and a pulse frequency of 80 kHz, 12 pulses overlap in each point; (**b**) Microscope image of P2 scribes at different fluence per pulse; (**c**) The image of the P2 ablation used for modules, in which a series of P2 scribes are done parallel to each other with a distance (RSD) of 10 µm. The red scale bars indicate 30 µm.

**Figure 3:**
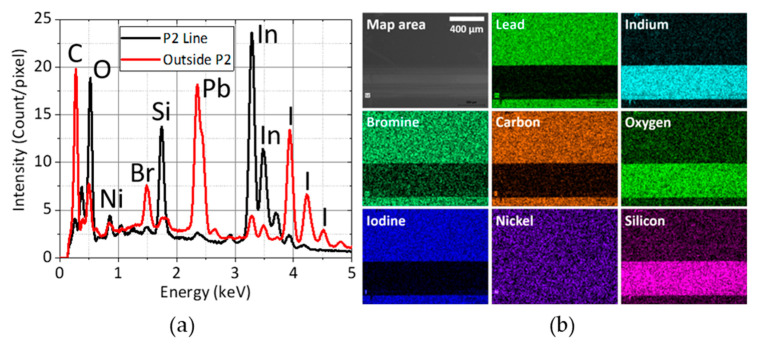
(**a**) Average EDX spectra over the P2 line and the area outside it; (**b**) A matrix of SEM image and EDX elemental maps. The P2 is located on the bottom side of each map.

The most common architecture used for perovskite modules is the n-i-p structure. It was used for the first modules fabricated back in 2014 with a PCE of 6.3% on 16.8 cm^2^ [14], and after years of research, it allowed the fabrication of a 21 cm^2^ module with a PCE of 18.13% in 2020 [15]. The upscaling of p-i-n cells can be more challenging due to the thin transport layers used, but a lot of progress has been made in recent years by using PTAA as an HTL. A record PCE of 19.1% (on AA, 17% on AR) was recently obtained using a combination of blade coating and thermal evaporation [6]. Compared to PTAA, there are only a few reports on inverted perovskite modules based on NiO_x_. We believe that NiO_x_ is a strong candidate for p-i-n modules due to its high stability [3], low cost, and lack of wetting issues which are often reported for organic HTLs such as PTAA [16]. There are also a variety of deposition techniques that can be used on large areas, ranging from spray coating to sputtering [17]. Liao et al. reported the first NiO_x_-based modules in 2017. They used a sol-gel ink to deposit the NiO_x_ layer on 5 × 5 cm^2^ substrates and used mechanical scribing with a blade for the P2 contact, obtaining an active area PCE of 12% [7]. During the same year Troughton et al. developed a process to implement the antisolvent deposition in air, fabricating modules with a PCE of 11.8% using mechanical scribing for the P2 contact [9]. Also the last report on NiO_x_-based modules made use of mechanical scribing, showing the lack of literature on the laser interconnection of this kind of module [8]. In this other work, Abzieher et al. developed the electron beam evaporation of NiO_x_ for highly efficient PSCs. Using thermal evaporation for the perovskite layer, as well as for the ETLs and the top contact, they obtained an all-evaporated minimodule of 2.3 cm^2^ area with a PCE of 12.4%. While mechanical scribing is also used in the industrial production of thin-film modules and it has been implemented in the fabrication of several perovskite modules [7,8,9,18], it has several disadvantages compared to laser ablation such as a large scribe width, chipping, and incompatibility with flexible substrates [19,20]. The evaluation of the P2 scribing process requires the understanding of the quality of the ablation: a qualitative evaluation can be achieved with a combination of optical microscopy and energy-dispersive X-ray spectroscopy (EDX) (see Figure 2 and Figure 3) [18]. On the other hand, the quantification of resistive losses expected in a P2 contact can be used to predict the impact on the module efficiency. This necessitates a transfer length measurement (TLM) in combination with electrical simulations. TLM allows the quantification of the contact resistance and introduces the concept of transfer length (L_T_) [21]. With this measurement we can properly simulate the losses in the P2 scribe. The cell-to-module losses can be separated into three contributions [22,23,24,25]: the resistive loss due to the electrode (in particular the TCO), the resistive loss in the P2 contact, and the area loss due to the interconnection. Besides, we should consider that the increase in the active area makes the modules more sensitive to defects, especially since in a series connection the total current at a given voltage is limited by the lowest among the different cells. The contribution of defects is not considered in the simulation, and it is evaluated by estimating the difference between the simulated module efficiency and the actual efficiency.

In this work, we compared electrical stimulation and experimental results to evaluate the shift from small cells to modules, with a particular focus on the laser interconnection. We demonstrated for the first time the use of UV laser ablation for NiO_x_-based modules and we estimated the resistive losses that arise from the size of the electrodes and the series interconnection as well as the geometric losses induced by the area dedicated to the interconnection. This enables a quantification of the total expected losses from those factors so that any additional losses can be addressed to defects generated during the fabrication procedure. We also provide an outlook that describes the possible evolution of perovskite modules and the best-case scenarios that we foresee.

## 2. Materials and Methods

Dimethylformamide (DMF-anhydrous-Sigma Aldrich), dimethyl sulfide (DMSO-anhydrous-Sigma Aldrich), 2-methoxyethanol (2-ME-anhydrous-Sigma Aldrich), chlorobenzene (CB-anhydrous-Sigma Aldrich), dichlorobenzene (DCB-anhydrous-Sigma Aldrich), Nitric acid (70%-Sigma Aldrich), isopropanol (IPA-anhydrous-Sigma Aldrich), Nickel chloride hexahydrate (NiCl∙6H_2_O-99.9%-Sigma Aldrich), [6,6]-phenyl-C_61_-butyric acid methyl ester (PCBM-99%-Solenne), Bathocuproine (BCP-96%-Sigma Aldrich), methylammonium bromide (MABr-99.99%-Greatcell solar), formamidinium iodide (FAI-99.99%-Greatcell solar), Cesium iodide (CsI-99.99%-Sigma Aldrich), Lead Bromide (PbBr_2_-TCI), Lead Iodide (PbI_2_-TCI), 1-butyl-3-methylimidazolium tetrafluoroborate (BMITFB-98%-ACROS) were purchased and used without further purification. Glass/ITO substrates (10 Ω □^−1^) were purchased from Kintec.

The nickel oxide ink was prepared by adding 35.5 mg of NiCl∙6H_2_O to 1 mL of 2-ME. After adding 20 µL of nitric acid the solution was heated at 75 °C for two hours [26]. The ink was aged for at least 2 days before use. The perovskite triple cation ink (Cs_0.05_MA_0.1425_FA_0.8075_PbI_2.7_Br_0.3_) was prepared by adding 1521.8 mg of PbI_2_, 104.3 mg of PbBr_2_, 44.8 mg of CsI, 479.1 mg of FAI and 55.0 mg of MABr to 1.899 mL of DMF and 0.601 mL of DMSO. The formulation contains a 4% excess of lead salts, and a DMF: DMSO volume ratio of 3.16:1. 50 µL of BMITFB were added to 1 mL of DMF [3], and 20 µL of this solution was added to 1 mL of the perovskite ink. Electron transport layers (ETLs) solution were prepared by adding 27 mg of PCBM to 750 µL of CB and 250 µL of DCB, and by adding 5 mg of BCP to 10 mL of IPA.

### 2.1. Cell Fabrication

2.5 × 2.5 cm^2^ glass/ITO samples were patterned with a UV ns laser (Spectraphysics - Andover, MA, USA) and diced with a glasscutter (Dyenamo – Stockholm, Sweden). Samples were scrubbed with water and soap solution (Hellmanex 2% in deionized water) and cleaned with three stages of ultrasonic bath: first in water and soap, then in ultrapure water, and finally in IPA. After drying they were treated for 15 min in a UV/O_3_ tool (Novasonic). The nickel oxide ink was spun at 4000 RPM for 30 s and annealed for 5 min at 75 °C, 10 min at 120 °C and one hour at 300 °C. After cooling down, the samples were transferred in a nitrogen-filled glovebox. The perovskite ink was spun at 4000 RPM for 35 s, and 180 µL of CB were dropped after 20 s. The film was annealed for 10 min at 100 °C. PCBM was spun at 1700 RPM for 30 s and annealed at 100 °C for 5 min. BCP was spun at 4000 RPM. 100 nm of gold were deposited by thermal evaporation, using a shadow mask to separate the four cells that are fabricated on each substrate.

### 2.2. Module Fabrication

**Figure 4:**
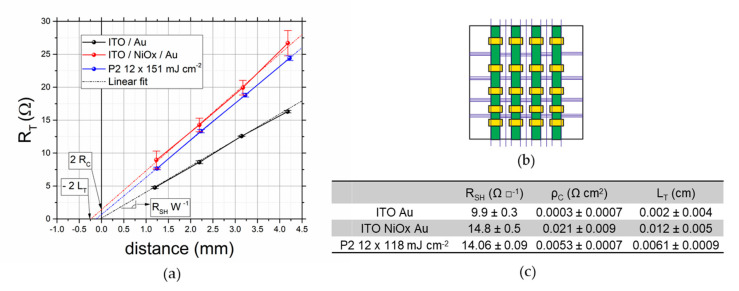
(**a**) Transfer length measurement (TLM) plot with an indication of the important parameters measure; (**b**) Design of the samples used for TLM: purple lines represent P1 scribes, yellow pads represent the gold contact and green pads indicate the four stripes used for TLM; (**c**) Summary table with the parameters measured in the TLM analysis.

5.6 × 5.6 cm^2^ glass/ITO samples were patterned and cleaned like small cells, and the same procedure was used to deposit all the layers (increasing the volume of ink and antisolvents used for spin coating of a factor of 3.5). The interconnection was made via a P1–P2–P3 scheme as shown in Figure 1. The optimal fluence per pulse (amount of energy of a single laser pulse divided for the area of the laser spot) was 280 mJ cm^−1^ for P1, 118 mJ cm^−1^ for the P2, and 90 mJ cm^−1^ for the P3. All the ablations were carried out from the top side of the device at a repetition rate of 80 kHz, with a scanning speed of 195 mm s^−1^. The patterns of the different scribes are shown in Figure 1. The P2 scribes were made after the deposition of all the layers up to the BCP, while the P3 scribes were made after the evaporation of the Au electrode. The samples for TLM followed the same procedures of the module, but the design is different (see Figure 4). No P3 was needed for TLM samples. The P2 ablation is made of a set of parallel scribes with an RSD (raster scan distance) of 10 µm. The P3 ablation is made of a set of parallel scribes with an RSD of 15 µm. The width of P1 is the one of a single scribe, in this case 15 µm. The width of P2 range from 60 to 300 µm (see last section of the results), while the width of P3 scribes is 105 µm. The cell width was equal to 5 mm and the interconnection width was fixed to 500 µm, and the P2 was centered between P1 and P3 scribes. For this reason, the distance among P2 and P2 or P2 and P3 was 40 µm for a P2 of 300 µm, 90 µm for a P2 of 200 µm, and 160 µm for a P2 of 60 µm.

### 2.3. Characterization

Cell and module were measured with an ABET Sun 2000 class A sun simulator, calibrated with an Eko MS-602 pyranometer. The accuracy of the calibration was checked by measuring the internal quantum efficiency of cells (Arkeo system from Cicci Research, Grosseto, Italy), ensuring that the mismatch of the integrated and measured J_SC_ was less than 4%. Data were acquired with a 4-channels source meter unit of Cicci Research with a scan rate of 100 mV s^−1^ for cells, 300 mV s^−1^ for modules with three cells, and 500 mV s^−1^ for modules with five cells. Resistance measurement of TLM samples was performed with a Keithley 2420 in 4-wire mode, using round copper probes. The absorbance of the NiO_x_ film was measured on glass microslides using a UV–Vis 2550 Spectrophotometer from Shimadzu (Kyoto, Japan).

Scanning electron microscopy (SEM) images and EDX elemental maps were acquired using a FEI Nova NanoSEM (ThermoFisher formerly FEI, Eindhoven, The Netherlands) fitted with a Bruker XFlash 6 EDX detector (Bruker Nano, Berlin, Germany). EDX maps and spectra were processed using Esprit 1.9 software. The current and voltage settings are 27 pA, 2 kV for imaging and 200 pA, 10 kV for EDX mapping. For cross-sectional TEM imaging, a lamella was prepared using a FEI Helios Nanolab Dualbeam FIB/SEM following a standard procedure [27]. This lamella was then immediately transferred into a FEI Osiris TEM operated in scanning mode (STEM) at 200 kV. The high-angle annular dark field (HAADF) image was acquired using a Fischione detector (Fischione, Hanau, Germany) with a dwell time of 1 µs/pixel.

## 3. Results

A first step to initiate an upscaling activity is the selection of a stack that can provide efficient and reproducible results. In this work we focused on a p-i-n architecture made of glass/ITO/NiO_x_/Cs_0.05_MA_0.1425_FA_0.8075_PbI_2.7_Br_0.3_+BMITFB/PCBM/BCP/Au (see Figure 1). The type of TCO and the addition of BMITFB ionic liquid were selected after a preliminary comparison (see Figure S1). The ionic liquid should also reduce the ion migration within the perovskite to prevent its degradation [3,28]. This type of PSCs showed PCEs between 16 and 17% with a low spread in the results, easing the fabrication of balanced modules. Since a module is made by a set of series-connected cells, the module’s current will be limited by the cell with the lowest current. Furthermore, due to the larger area and more complex fabrication, the modules often come with a lower set of samples produced. The strategy selected for the series connection is the fabrication of a P1–P2–P3 interconnection by performing three laser ablation processes with a ns UV laser. The P1–P2–P3 scheme is shown in Figure 1.

In this work, we fabricated three types of PV devices: small PSCs with four 0.09 cm^2^ active areas on 2.5 × 2.5 cm^2^ substrates, 3-cell minimodules with an active area of 2.25 cm^2^ on 2.5 × 2.5 cm^2^ substrates, and 5-cell minimodules with an active area of 10.2 cm^2^ on 5.6 × 5.6 cm^2^ substrates (see Figure 1). The smaller minimodules were used to optimize the fabrication of the interconnection since they can be fabricated in larger numbers (improving the statistical relevance of the results) and do not require changes in the layer deposition protocol when compared to small cells. Besides the selection of the stack and proper design of the module, to enable the fabrication of modules it is necessary to develop the laser scribing processes. P1 and P3 scribes are used to electrically separate the electrodes of adjacent cells. The P1 scribing of glass/TCO samples is usually the simplest one and does not require specific investigation. A single laser line with a width of 15 µm was sufficient to provide insulation among different areas of the ITO (Figure S2). The P3 scribe is more challenging since the layers underneath the top metal electrode may be damaged by parasitic heating induced by the laser pulses [29]. Nevertheless, it was possible to successfully transfer the P3 process for gold electrodes as developed in previous studies, obtaining good electrical insulation with a P3 width ranging from 90 to 150 µm [30]. The main focus in the development of an efficient interconnection was on the optimization of the P2 scribe. The P2 should remove all the layers from the bottom transport layer (here NiO_x_) up to the top transport layer (here BCP) without damaging the bottom electrode. The UV wavelength used (355 nm) is absorbed by all the layers of the stack, including ITO. This requires careful tuning of the laser fluence to optimize the trade-off between incomplete removal of the layers at low fluences and ITO damage at high fluences. In particular, the laser is only partially absorbed by the NiO_x_ layer (see Figure S3), suggesting that it might be challenging to achieve a selective removal of all the NiO_x_ without arming the ITO. An optical image of a power scan is reported in Figure 2.

At low fluences per pulse (amount of energy of a single laser pulse divided for the area of the laser spot, <70 mJ cm^−2^ pulse^−1^) we can observe a narrow ablation line. In these cases, the fluence is above the ablation threshold only at the center of the laser beam: the power density of the laser beam typically has a Gaussian shape and, in these images, we can only observe the width of the ablated material. The yellow color of the reflected light also suggests the incomplete removal of the layers. This is supported by the yellow color observed at the side of each P2 scribe even at higher fluences: at the side of each scribe the ablation is incomplete due to the Gaussian shape of the intensity of the laser spot. At high fluences, we can observe the cracking of the ITO layer (fluence > 130 mJ cm^−2^ pulse^−1^) or its complete ablation in the center of the scribe (fluence > 190 mJ cm^−2^ pulse^−1^). The optimal fluence lies in between these two boundaries (poor ablation and ITO cracking). Furthermore, to provide a sufficient width of the P2 contact, we used a set of parallel scribes at a raster scanning distance (RSD) of 10 µm (see top right panel of Figure 2). A P2 scribe with a fluence of 118 mJ cm^−2^ pulse^−1^ and an RSD of 10 µm was selected for in-depth characterization. It is important to underline that the optimization of the P2 scribe with optical microscopy (i.e., by observing the color of the different areas of the scribe) is eased by the low roughness of the ITO electrode that gives rise to interference contrast, enabling the detection of thin transparent layers by observing the color of the image. We believe that a similar analysis would be more challenging for a rougher TCO such as fluorine-doped tin oxide.

Figure 3 shows the top-view EDX analysis of the P2 scribe and the area surrounding it. The perovskite-forming elements (C, Pb, I, and Br) are removed from the P2 scribe with excellent selectivity without removing the ITO layer. However, the removal of NiO_x_ is incomplete as indicated by the small nickel peak at 0.85 keV in the P2 Line spectrum. Ideally, the NiO_x_ layer should be entirely removed to minimize series resistance. However, due to the NiO_x_ layer’s low thickness (about 15 nm) and the lack of selectivity of the UV laser, it is very challenging to remove it completely without damaging the ITO layer. We note that although the EDX spectra and the nickel map in Figure 3 seem to indicate that there is the same amount of nickel both inside and outside the P2 line, this is actually not the case. There is far less nickel inside the P2 line as outside P2, approximately 91% of the generated Ni-Lα X-ray photons were absorbed by the perovskite layer and hence did not reach the EDX detector (see Figure S4). On the other hand, NiO_x_ is the topmost layer inside the P2 line so effectively all the Ni-Lα X-ray photons generated there were able to escape from the sample surface.

To understand the impact of the incomplete removal of NiO_x_, we performed a TLM analysis of the contacts. In this way, we can quantify the quality of the P2, and we can use the results to simulate the resistive losses that can be expected. The TLM is required to have a proper estimation of the resistive losses since the high aspect ratio of a thin-film contact (width of the contact over the thickness of layers) prevents a more direct measurement [21].

The geometry of this kind of contact (metal over a less conductive thin semiconductor) is characterized by a high aspect ratio among the width of the contact and the thickness of the electrode, inducing current crowding on one side of the contact: the current flowing on one edge of the contact is high, and its density decays in an exponential fashion with a characteristic length L_T_. In other words, the effective contact length can be much smaller than the actual contact length, and there is no need to exceed L_T_ when defining the optimal width of a P2 contact. For this reason, L_T_ is a more significant parameter than the contact resistance to calculate the efficiency of a P2 contact, and it is needed to perform a proper electrical simulation of a module. We can consider L_T_ as the length that the charges travel in the TCO before they are transferred to the contact. It is related to both specific contact resistance (ρ_C_) and the conductivity of the electrode (R_SH_) with the following equation:

(1) $$L_{T}=\;\sqrt{\frac{\rho_{C}}{R_{\mathrm{SH}}}}\;.\;$$

With this definition, we can express the total resistance (R_T_) measured with TLM as:

(2) $$R_{T}=\frac{R_{\mathrm{SH}}}{W}\left(L+L_{T}\right)\;,$$

where W is the width (perpendicular to the current flow) of the contact and L is the distance between contacts. A summary of the TLM results is shown in Figure 4.

**Figure 5:**
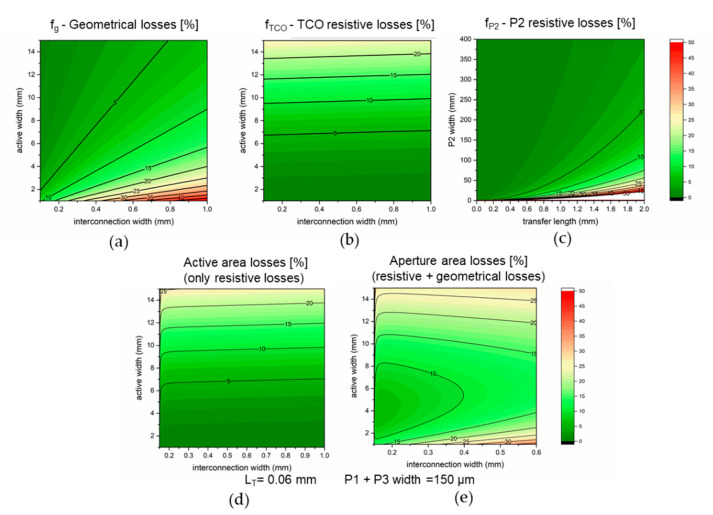
Breakdown of the relative cell-to-module efficiency losses. (**a**) Geometrical losses due to the presence of the interconnection; (**b**) The resistive losses due to the TCO; (**c**) The resistive losses due to the P2 contact; (**d**) The losses expected on active area efficiency; (**e**) The losses expected on aperture area efficiency. The TCO considered here has a R_SH_ of 15 Ω □^−1^, J_MPP_ is 19 mA cm^−2^ and V_MPP_ is 850 mV.

As expected, the lowest transfer length is obtained with an ITO/Au contact without any interlayer and the results obtained are in line with the ones already presented in the literature [24]. Interestingly, the sheet resistance of the TCO increase from 10 to about 15 Ω □^−1^ after the deposition and annealing of the NiO_x_. 4-point probe measurement reveals that the sheet resistance does not increase by heating the bare ITO to 300 °C, so the increase in resistivity is due to the deposition of NiO_x_. We speculate that the acidity of the sol-gel or the migration of In to the NiO_x_ can be the reasons behind this phenomenon. The presence of NiO_x_ in between ITO and Au increases the transfer length from 0.002 to 0.012 cm (increase in ρ_c_ from 0.0003 to 0.02 Ω cm^2^). While this is a considerable increase, the impact on the cell-to-module losses is almost negligible if one uses a P2 width of more than 100 µm (see next section). The L_T_ measured for the solar cell stack after the laser ablation discussed above (shown in Figure 2) is considerably lower, thanks to the reduction of ρ_C_. This measurement confirms that the laser ablation process can partially remove the NiO_x_ layer. The remaining NiO_x_ (as also observed in the EDX) justify the higher contact resistance compared to a direct ITO/Au contact. To understand if the proposed P2 ablation process is sufficient to minimize the cell-to-module losses it is necessary to break down the loss factors. We implemented a model developed for thin film-modules (and already used for perovskite PV) that separate the losses into three factors [22,23,24,25]: the geometrical losses due to the dead area of the P1-P2-P3 interconnection (f_G_), the resistive losses due to the TCO (f_TCO_), and the resistive losses due to the P2 contact (f_P2_). These are calculated using the following equations:

(3) $$f_{G}=\frac{w_{\mathrm{int}.}}{w_{\mathrm{int}.}+w_{\mathrm{active}}},$$

(4) $${\;f}_{\mathrm{TCO}}=\frac{J_{\mathrm{MPP}}}{V_{\mathrm{MPP}}}\cdot \frac{R_{\mathrm{SH}}^{\mathrm{TCO}}}{3}\cdot \frac{w_{\mathrm{active}}^{3}}{w_{\mathrm{active}}+w_{\mathrm{int}.}}\;,$$

(5) $${\;f}_{P2}=\frac{J_{\mathrm{MPP}}}{V_{\mathrm{MPP}}}\cdot \frac{w_{\mathrm{active}}^{2}}{w_{\mathrm{active}}+w_{\mathrm{int}.}}\cdot L_{T}{\;R}_{\mathrm{SH}}^{P2}\coth\frac{w_{P2}}{L_{T}}\;,$$

where w_int_ is the width of the interconnection (from P1 to P3 of the same interconnection), w_active_ is the active width of the cell (from one P3 to the P1 of the next interconnection), w_P2_ is the width of the P2 scribe, R_SH_ is the sheet resistance (in the TCO or the P2), J_MPP_ is the maximum power point current density and V_MPP_ is the maximum power point voltage. The trends of the different functions are plotted in Figure 5.

**Figure 6:**
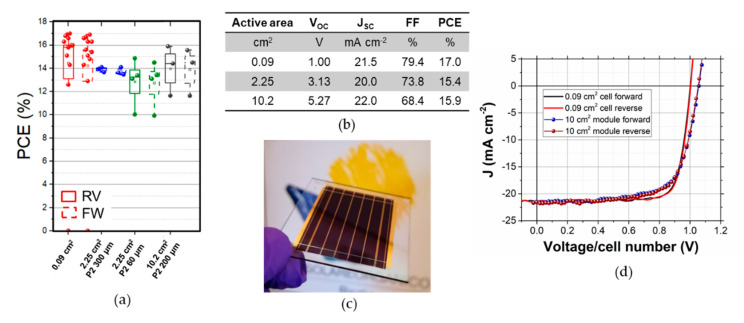
(**a**) Box plot of the active area efficiencies of the devices fabricated for this work; (**b**) Summary table with the PV parameter of the best device per each size; (**c**) Picture of the 10.2 cm^2^ module; (**d**) JV curve (voltage is divided by number of cells) of the best 0.09 and 10.2 cm^2^ device.

If we focus on the losses due to the P2 scribe, we can observe that with a L_T_ of 0.06 mm any w_P2_ above 10 µm will result in resistive losses lower than 0.1%, meaning that the P2 ablation developed is well-suited to make inverted modules with high efficiency. Even without removing the NiO_x_ (L_T_ = 0.2 mm), it is possible to have rather low losses, i.e., a loss of 0.2% with a w_P2_ of 10 µm or a loss of 0.1% with a w_P2_ of 120 µm. The other losses are affected by the resistivity of the TCO and the design of the module (i.e., w_active_ and w_int._). If one wants to maximize the active area efficiency of the module, only the resistive losses will matter (f_TCO_ and f_P2_), while the geometrical losses should be considered only in the aperture area efficiency. To understand the expected losses on the module, we fixed the L_T_ to the one obtained with our laser ablation, and we calculated the interconnection width as w_P2_ plus 150 µm for the P1 and P3. To maximize the active area efficiency, it is important to minimize w_active_ to reduce the resistive losses in the TCO, while the impact of the interconnection is negligible whenever the P2 is wider than 15 µm. This is true only if the L_T_ of the P2 contact is low enough to prevent large losses in the P2 contact, otherwise w_P2_ would have a larger impact also on the active area PCE [22]. Given L_T_ obtained with our P2 ablation, we do not expect any difference in modules with w_P2_ ranging from 60 to 300 µm. To have experimental confirmation of this calculation we fabricated minimodules with three cells connected in series for a total active area of 2.25 cm^2^. In Figure 6 we can observe that there is a negligible difference among minimodules with P2 of 300 or 60 µm in width.

The larger spread when using w_P2_ of 60 µm can be due to local disconformity in the layers that lead to a longer L_T_ or to extrinsic factors. This suggests that widening the P2 could lead to a more robust process without any negative impact on the active area PCE. Furthermore, the 2.25 cm^2^ modules have a J_SC_ lower than the cell, most likely due to an unbalance among cells. We speculate that the antisolvent washing used for the perovskite layer is not uniform in the center of the sample, and this issue can have a noticeable impact due to the small size of the cells. When 10.2 cm^2^ modules were fabricated the J_SC_ was in line with one of the small cells. By increasing the w_P2_ to 120 µm we aimed to mitigate any issue related to disuniformity in the P2 ablation process. Given the larger area of the devices and the lower number of samples, it is crucial to take all the precautions to increase the yield of the process. In this way, we demonstrated the fabrication of inverted modules with NiO_x_ with an active area efficiency of 15.9% (14.5% on aperture area, with a geometrical fill factor of 90.9%), the highest for a NiO_x_-based perovskite module (calibration verified via IPCE of a small cell, see Figure S5). If we consider that the w_active_ is 5 mm and the w_int._ is 500 µm, we would expect a cell-to-module loss of 3%. It is worth noticing that for a fairer comparison, we should exclude the resistive losses included in the measurement of the small cell, as well as the loss in V_OC_ due to the use of illumination masks (see Figure S6) [31]. Even if the lack of mask would likely induce the reduction of FF, this reduction should not be considered to evaluate the cell-to-module losses since it is specific for a given cell layout [32]. If we include these corrections, we should increase the PCE of the small cells of a factor of 4%, leading to modules with potential active area efficiency of 17.2%. The additional losses (7.5% relative) may be attributed to the increase of defects that are often related to any upscaling activity. Indeed, if we consider the average efficiency of the small cells (16.5% after the corrections mentioned above), the potential active area efficiency of the module would be 16%. This confirms that the losses observed are in line with reasonable expectations and that improving the reproducibility of the process is a key requirement for any improvement in the upscaling activities. The last plot of Figure 5 shows the expected losses on the aperture area, which is important to define the energy yield of a PV module. There we can observe how critical the miniaturization of the P2 scribe is and the need for a low L_T_, since the cell-to-module losses can be lower than 6% only with a cell width of 4 mm and an interconnection width of 166 µm. We can observe the impact of L_T_ on the aperture area PCE with the simulation presented in Figure S7: increasing L_T_ to 0.2 mm limit the minimum loss to 7.5% due to the widening of w_P2_ that increase the geometrical losses. Besides improving the laser ablation process (i.e., reducing safety areas or the width of P1 and P3), a significant reduction of the losses can be achieved only by lowering the R_SH_ of the TCO or by minimizing the J_MMP_ to V_MPP_ ratio (see Equation (4) and (5)). For the former, we can simulate the aperture area PCE losses when using a TCO with 7 Ω □^−1^, a value that can be easily obtained by using fluorine-doped tin oxide. With this type of TCO we can lower the minimum aperture area PCE loss to 4.6% thanks to a decrease in the resistive loss in the electrode that allows for wider cells. This might not result in an improvement of the overall efficiency due to the reduced transparency of more conductive TCO. Reducing R_SH_ below 7 Ω □^−1^ requires the addition of collecting grids: in this case, the improvement in R_SH_ must be balanced with the absorption losses due to the shadowing of the grid. We believe that the improvement introduced by grids for series-connected modules would be minor, while they will be essential for large area cells (i.e., for the front contact of a Si/perovskite tandem). Once both L_T_ and R_SH_ are minimized the only factor that can be tuned is the J_MMP_ to V_MPP_ ratio. The ratio used in our simulation would not change significantly even considering highly efficient single-junction PSCs, since they would increase both J_MMP_ and V_MPP_. To achieve a significant lowering of these parameters we must consider the use of two-junction or three-junction tandems [33]. All-perovskite tandems are an interesting approach to go beyond the efficiency limit for a single junction PSC and using them for perovskite modules would simultaneously lower the J_MPP_ and increase the V_MPP_. When we simulate the cell-to-module losses for a two-junction tandem cell with PCE of 27% and a three-junction tandem cell with PCE of 30.8% (J_MPP_ and V_MPP_ used are reported in the table of Figure S7) we can observe that the expected losses decrease to 3.3% and 2.6% respectively (corresponding to modules with aperture area PCE of 26.1% and 30%). The results indicate how the increase of PCE obtained with tandem structures compared to single junction PSC will be amplified during the upscaling from cell to modules.

## 4. Conclusions

In conclusion, we demonstrated that is possible to obtain an efficient P2 ablation of inverted PSCs with NiO_x_ using a ns UV laser. This is the first published report of a UV laser ablation process for a NiO_x_-based module. A large part of the optimization was carried out with optical microscopy, while SEM-EDX showed that the removal of NiO_x_ is incomplete. The P2 contact was also studied by a TLM analysis to quantify the quality of the contact by measuring the transfer length L_T_. By knowing this factor, we simulated the cell-to-module losses to better evaluate the results obtained during the upscaling study. The validity of the estimation of the resistive losses was also verified experimentally by varying the width of P2 in 2.25 cm^2^ modules, confirming the prediction of the simulations. With the optimized P2, we were able to fabricate a 10.2 cm^2^ module with an active area PCE of 15.9%, only 6.5% lower than the best results on small cells. This represents the highest efficiency for a perovskite module using NiO_x_ as the HTL. Since the expected losses on active area were equal to 3%, the efficiency of the module is in line with the average PCE of small cells. To further improve this structure, it is required to increase the reproducibility of the processes (improving the fabrication protocol or by using a stack that is less sensitive to defects) and improve the overall PCE of the cells. From the theoretical analysis of the losses, we can suggest that further reduction in the cell-to-module losses on aperture area can be achieved by minimizing the width of the interconnection, by lowering the sheet resistance of the TCO and by moving to a tandem structure, where the losses can be as low as 2.6% for a three-junction device.

## Supplementary Materials

The following are available online at https://www.mdpi.com/2072-666X/11/12/1127/s1, Figure S1: box plots of the PV parameters of pin solar cells with different TCOs and with or without the addition of BMITFB. Figure S2: picture of a P1 P2 P3 interconnection. Figure S3: absorbance spectra of the NiO_x_ layer. Figure S4: simulation of the absorbed and emitted EDX photons of the NiO_x_. Figure S5: IPCE measurement of the stack used for module fabrication. Figure S6: effect of mas on the JV measurement of small cells. Figure S7: Additional simulation of cell-to-module losses.
