# Supplementary material for: Upscaling Inverted Perovskite Solar Cells: Optimization of Laser Scribing for Highly Efficient Mini-Modules
Source: Micromachines (Basel). 2020 Dec 20;11(12):1127. doi: 10.3390/mi11121127 (PMC7767295; doi:10.3390/mi11121127)
Supplement: Supplementary file 1 [file micromachines-11-01127-s001.pdf]

micromachines

Supplementary Materials: Upscaling Inverted Perovskite Solar Cells: Optimization of Laser Scribing for Highly Efficient Mini-Modules

Francesco Di Giacomo [1,*], Luigi A. Castriotta [1], Felix U. Kosasih [2], Diego Di Girolamo [3], Caterina Ducati [2] and Aldo Di Carlo [1,4,*]

[1] Centre for Hybrid and Organic Solar Energy (CHOSE), Department of Electronic Engineering, University of Rome Tor Vergata, 00133 Rome, Italy; luigi.angelo.castriotta@uniroma2.it
[2] Department of Materials Science and Metallurgy, University of Cambridge, 27 Charles Babbage Road, Cambridge CB3 0FS, UK; fuk21@cam.ac.uk (F.U.K.); cd251@cam.ac.uk (C.D.)
[3] Department of Chemical Materials and Production Engineering, University if Naples Federico II, Piazzale Tecchio 80, Fuorigrotta, 80125 Naples, Italy; diego.digirolamo91@gmail.com
[4] LASE–Laboratory for Advanced Solar Energy, National University of Science and Technology MISiS, Leninsky Ave. 6, 119049 Moscow, Russia
* Correspondence: francesco.di.giacomo@uniroma2.it (F.D.G.); aldo.dicarlo@uniroma2.it (A.D.C.)

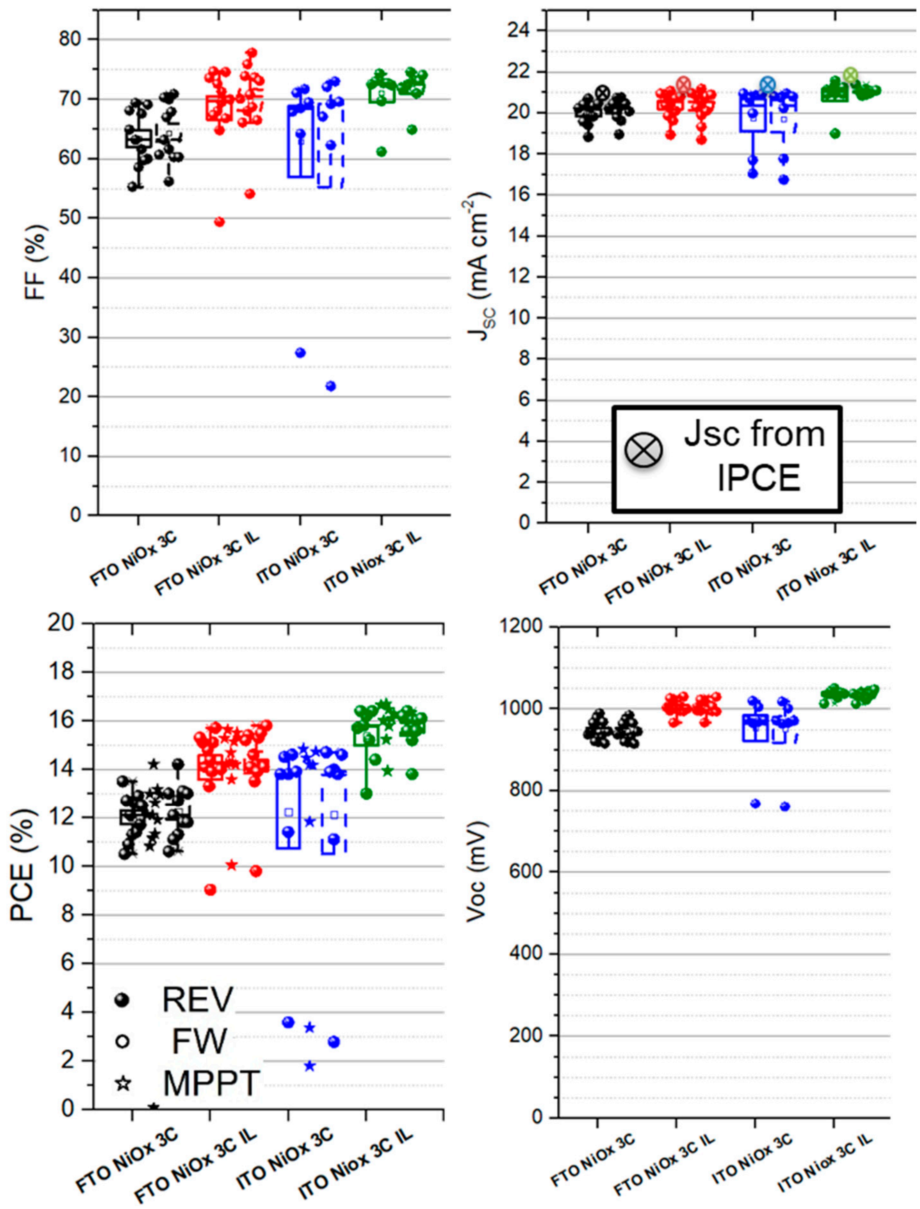

Figure S1. box plots of the PV parameters of pin solar cells with different TCOs (FTO or ITO) and with or without the addition of BMITFB (indicated as IL).

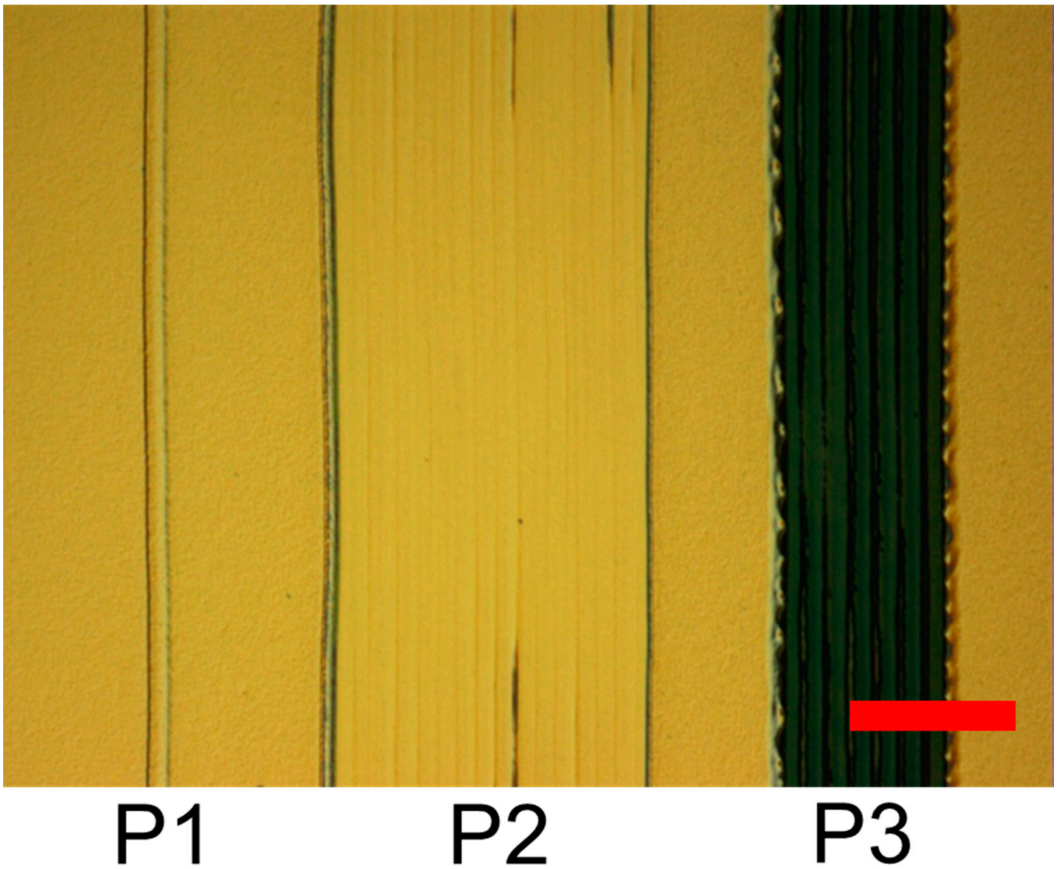

Figure S2. an optical image of a complete P1 P2 P3 interconnection used for the 10.2 cm^2 modules (total width is 500 μm). In this case, the width of P1, P2, and P3 scribes are 15, 200, and 110 μm respectively. The red scale bar represents 100 μm.

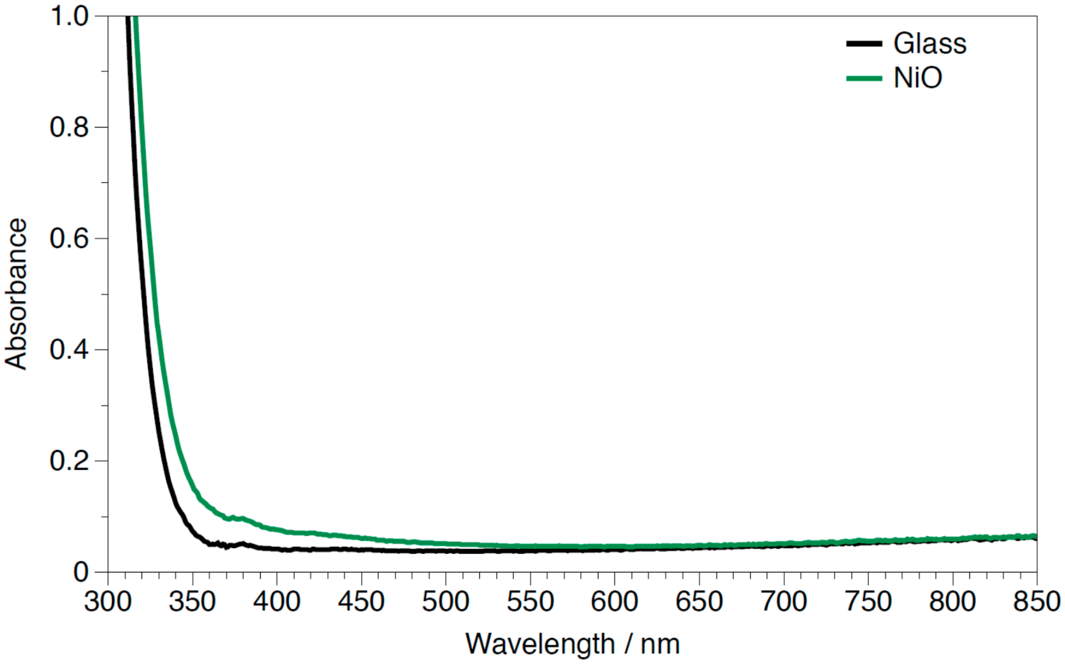

Figure S3. absorbance spectra of a NiO_x film on glass. The absorbance of pristine glass is presented to show the increase of absorbance due to the NiO_x. The thickness of the NiO_x was largely increased (by about a factor of 9) to enhance the absorbance.

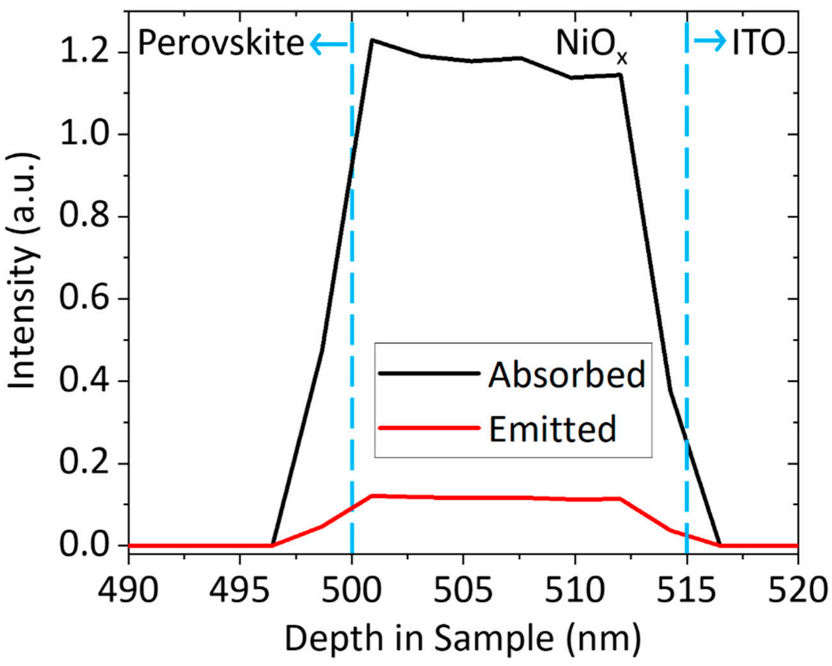

Figure S4. Simulated profile of Ni-L$_\alpha$ X-ray photon generation for the EDX data shown in Figure 3. The red plot shows the share of Ni-L$_\alpha$ X-ray photons which were emitted from the sample surface, while the black plot shows those which were absorbed inside the sample. Blue dashed lines mark the position of NiO$_x$ layer in the device stack, which is composed of glass/ITO (250 nm)/NiO$_x$ (15 nm)/perovskite (500 nm). The thicknesses of the top three layers are based on the cross-sectional STEM image shown in Figure 1.

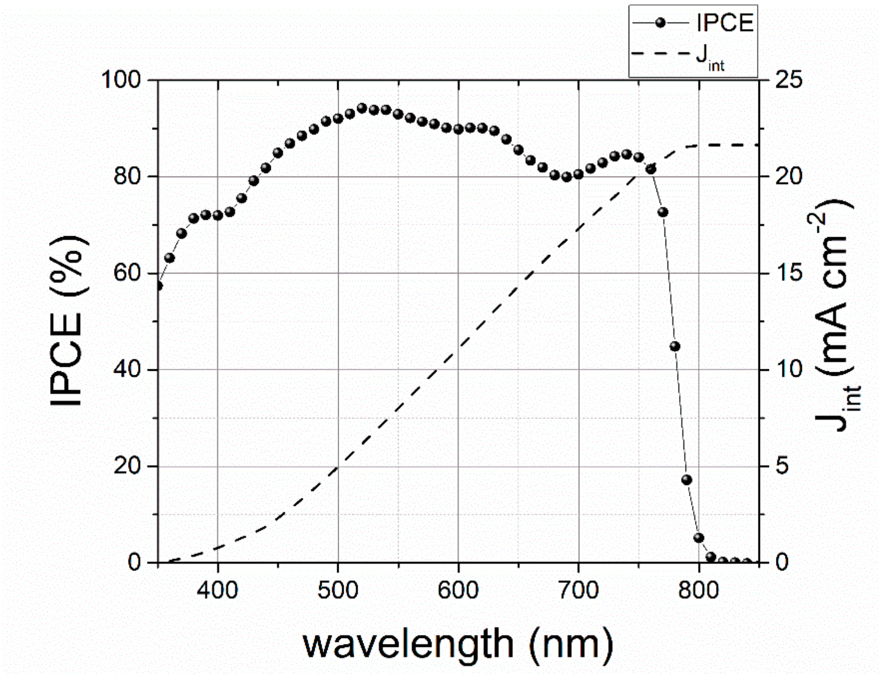

Figure S5. IPCE measurement and integrated J_{SC} for a representative small cell fabricated in parallel to the modules. The integrated J_{SC} is well below a 3% difference with the J_{SC} measured under sun simulators.

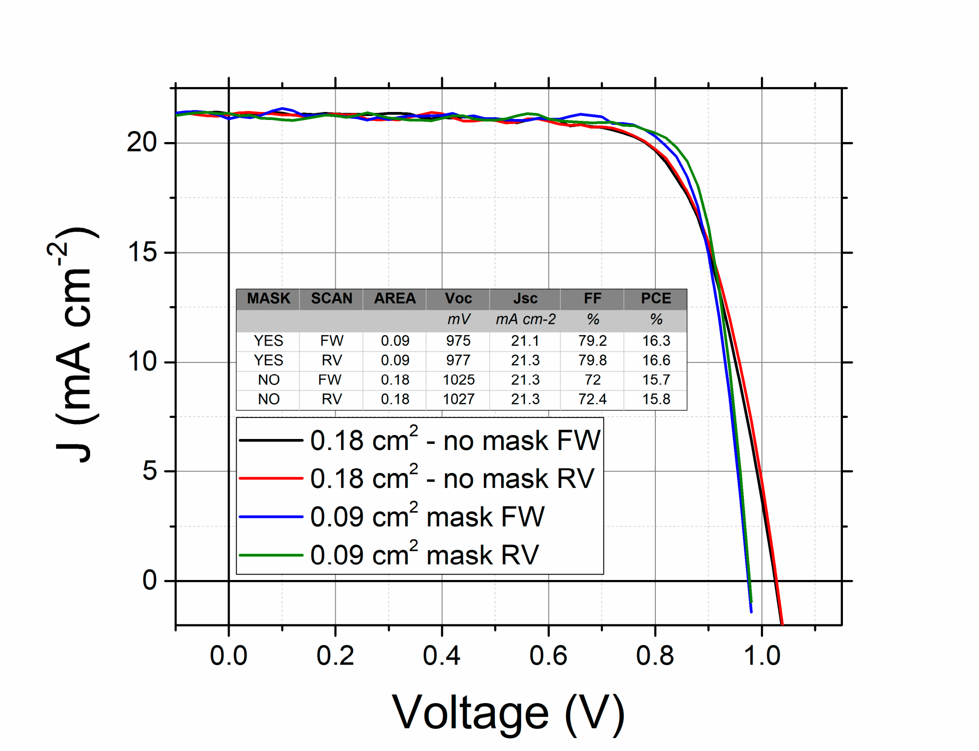

Figure S6. plot of JV measurement of a small cell with and without mask. We can observe how the use of a mask can reduce the Voc of about 50 mV, while it can increase the FF by reducing the Isc and the resistive losses associated with it.

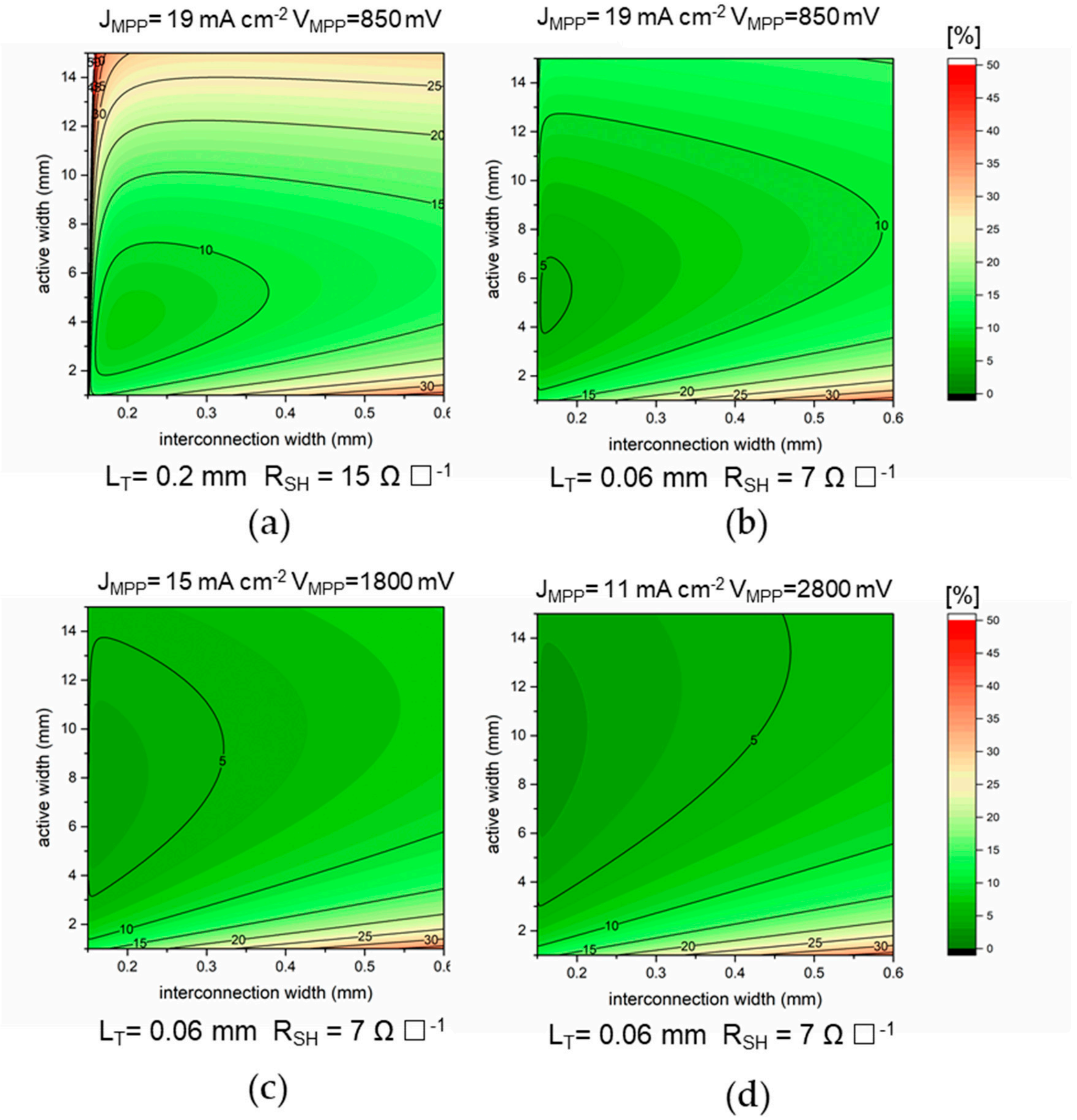

L_T	R_{SH}	J_{MPP}/V_{MPP}	Min loss Aperture area	w_{active}	$w_{int.}$
[mm]	[Ω□$^{-1}$]	[mA cm^{-2} mV^{-1}]	[%]	[mm]	[mm]
0.06	15	19/850	6	3.9	0.166
0.2	15	19/850	7.5	4.1	0.2
0.06	7	19/850	4.6	5.1	0.16
0.06	7	15/1800	3.3	7.8	0.16
0.06	7	11/2800	2.6	9.4	0.16

(e)

Figure S7. plot of the cell to module losses calculated on the aperture area to show the effect of tuning the parameters used for the simulation, as the L_T, the R_{SH}, and the ratio among J_{MPP} and V_{MPP}. (**a**) Effect of a higher LT of 0.2 mm compared to the one used in Figure 5; (**b**) the effect of a lower R_{SH}; (**c**) Simulation of a module based on a 2-junction perovskite tandem with a PCE of 27%; (**d**) Simulation of a module based on a 3-junction perovskite tandem with a PCE of 30.3%; (**e**) Summary table.
